# The impact of gender and academic achievement on the violation of academic integrity for medical faculty students, a descriptive cross-sectional survey study

**DOI:** 10.1186/s12909-019-1865-7

**Published:** 2019-11-20

**Authors:** Müesser Özcan, Neşe Yeniçeri, Edip Güvenç Çekiç

**Affiliations:** 1 The Faculty of Medicine, Department of Medical History and Ethics, Muğla Sıtkı Koçman University, Kötekli Mahallesi Marmaris Yolu Bulvarı No:50, 48000 Menteşe, Muğla Turkey; 2 The Faculty of Medicine, Department of Family Medicine, Muğla Sıtkı Koçman University, Kötekli Mahallesi Marmaris Yolu Bulvarı No:50, 48000 Menteşe, Muğla Turkey; 3 The Faculty of Medicine, Department of Medical Pharmacology, Muğla Sıtkı Koçman University, Kötekli Mahallesi Marmaris Yolu Bulvarı No:50, 48000 Menteşe, Muğla Turkey

**Keywords:** Academic integrity, Medical students, Dishonesty

## Abstract

**Purpose:**

The aim of this study is to determine the characteristics of medical faculty students about violations of academic integrity.

**Method:**

From the whole population of the 572 students of the Muğla Sıtkı Koçman University Faculty of Medicine, 271 students participated voluntarily in a descriptive cross-sectional survey. Descriptive data were recorded in the survey and a five-point Likert-type instrument, namely the Tendency towards Academic Dishonesty Scale, was used as the data collection tool in the study. The scale included 22 items’ means that are considered to evaluate “Tendency towards academic dishonesty” (TTAD) score. In addition, four subscales, namely “Tendency towards cheating”, “Dishonesty in works such as assignments and projects”, “Tendency towards dishonesty in research and reporting processes” and “Tendency towards citation dishonesty” scores were evaluated separately.

**Results:**

Of the participants, 138 (53.3%) were male. TTAD scores were 2.15 ± 0.61, showing a slight tendency towards academic dishonesty, according to the scale. TTAD scores and standard deviations (SD) were 2.26 ± 0.65 and 2.04 ± 0.55 for men and women, respectively (*P* = 0.005). There was no difference in the TTAD scores for students whether they had read the ethics code. Significant differences were observed in the TTAD scores for students with gender, different academic achievements and in different academic years. However, when multivariate analysis was performed, the significance shown in the results disappeared.

**Conclusion:**

In our study, a slight tendency to academic dishonesty was found for medical faculty students and there were no differences between all of the recorded individual factors of students.

## Introduction and background

Academic dishonesty is defined as wilful fraud or dishonest behaviour displayed when fulfilling academic requirements [1]. Behaviour such as copying or using the work of another individual without his/her permission, attending an exam in place of another individual, cheating in an exam, exchanging exam papers or taking exam papers outside the exam hall, violating the rules of exams and assaulting academic personnel verbally or physically is defined as academic dishonesty [2, 3].

Particularly because of the rapid advances in technology, academic dishonesty has become more common among university students [4–6].

Medical education should produce talented and qualified physicians, and students should be expected to convert the virtues of the medical profession, into personal character traits at the end of their theoretical and practical courses. Internationally accepted virtues of the medical community dating back thousands of years are as follows: *1) Trustworthiness 2) Compassion 3) Prudence 4) Justice 5) Temperance 6) Fortitude 7) Self-Effacement* and 8) *Integrity* [7, 8].

Therefore, wrongful academic actions by medical students’ conflict with the behaviours expected of a good physician [9–11].

They may also cause prospective physicians to be deprived of the necessary scientific knowledge, and deviate from one of the most important values of the medical profession, which is honesty [6]. Tiong et al. revealed that there was a significantly higher prevalence of academic dishonesty among healthcare academics than among their peers who did not work in healthcare [12]. In research conducted in Coratia, only 9% of 198 sophomores stated that they never plagiarized at all [6]. The academic misconduct of students is acquiring a different structure with the development of technology, and this is an important issue for discussion in medical education [10, 13–16]. For example a case of exam cheating at an Australian medical school happened in 2013. The students were reported to using tablet computers during the exam and this was covered by both local and international media. After this case, it was opened to discussion that these unprofessional behaviors of medical students should be unacceptable because of the characteristics of medical profession [10].

In literature, there are many studies on the academic dishonesty of medical students; there are some studies on the academic dishonesty levels of nursing students in Turkey [17, 18] however there are no such study conducted for medical students.

The aim of this study is to determine the attitudes and characteristics of medical faculty students in relation to the violation of academic integrity.

## Methods

### Participants and characteristics

The population of the study consisted of 572 undergraduate students who were attending courses in the Faculty of Medicine at Muğla Sıtkı Koçman University, Turkey. Students participated voluntarily with a non-random sampling method in the descriptive cross-sectional survey in response to verbal invitations given in lecture breaks between the months of April and June 2018. The 259 subjects who completed the entire survey were included in the study. The only exclusion criterion in this study was not to have completed the descriptive data questions completely.

Descriptive data (gender, medical academic year, academic achievement and whether they read the ethics code) were recorded. Academic achievement was measured by the answer to the question “What is your academic achievement level?” by categorizing themselves as “below the class academic average”, “approximately at the class academic average” and “above the class academic average”. In addition, the participants of the study were asked: “Have you read the Student Disciplinary Regulation for Higher Education Institutions? (ethics code)”.

### Instrument and procedures

After the descriptive data, the Tendency towards Academic Dishonesty Scale was used as the assessment tool. There are 22 items in this scale, which was prepared by Eminoğlu et al. in Turkish following validity–reliability study [19]. There were four basic subscales are assessed [19]: (1) “Tendency towards cheating: five items; one negative and four positive”, (2)“Dishonesty in works such as assignments and projects: seven items; four negative and three positive”, (3)“Tendency towards dishonesty in research and reporting processes: four items; one negative and three positive” and (4)“Tendency towards citation dishonesty: six items: three negative and three positive”. A total of nine negative and thirteen positive items are measured. The answers given to all items in the scale are digitized from five to one for the positive items and from one to five for the negative items. The Cronbach alpha internal consistency reliability coefficient of the whole scale was reported as 0.90, and the internal consistency reliability coefficients of the subscales were reported as 0.71, 0.821, 0.785 and 0.766 for the first factor to the fourth factor [19]. The test–retest reliability coefficient obtained as a result of applying the scale twice to a group of 20 people at 15-day intervals was reported as 0.88 [19].

The values of the means and standard deviations (SD) for the participants were calculated for each subscale and for the scale in general. A value between 1.00 and 1.79 is considered as a low tendency towards academic dishonesty; a value between 1.80 and 2.59 is considered as a slight tendency towards academic dishonesty; a value between 2.60 and 3.39 is considered as a moderate tendency towards academic dishonesty; a value between 3.40 and 4.19 is considered as a high tendency towards academic dishonesty; and a value between 4.20 and 5.00 is considered as an extremely high tendency towards academic dishonesty [19]. The values were re-assessed by dividing the students into groups based on their gender, academic year and academic achievement, and on whether or not they had read the ethics regulation, to show that there was no difference among the groups.

### Data analysis

All answers were recorded on a computer with a paper-based optical reader. The mean and SD for subscales were calculated as shown by Eminoğlu [19]. Pearson’s chi square test was used to calculate the differences between gender in different characteristics (academic year, academic achievement and having read the ethics code). The independent samples T test was applied to evaluate the differences of scale scores between gender and having read the ethics code. One-way ANOVA test was applied to evaluate the differences for academic year and academic achievement and post hoc Tukey test was applied as multiple comparison when assessing the intergroup differences of the participants. An univariate multivariable analysis was applied for assessing the relationship between the tendency towards academic dishonesty score and fixed factors of gender, academic achievement and academic year. A value of *P* of less than 0.05 was considered to be significant. Microsoft Excel (2007) software was used for recording the data on the computer, and the IBM SPSS Statistics for Windows, Version 20.0. Armonk, NY: IBM Corp program package was used for the statistical analyses. The study was approved by the Muğla Sıtkı Koçman Human Research Ethics Committee (approval date and number: 2018 / 43).

## Results

A total of 271 students (response rate: 47.3%) participated voluntarily in the study. In our study, the Cronbach alpha internal consistency reliability coefficient was calculated as 0.892 for the whole scale (22 items). This value indicates that there is coherence and a high rate of reliability throughout the scale. The characteristics of the population are given in Table 1.

**Table 1:** Characteristics of the population

Characteristics		n (column %)	Male n (row %)	Female n (row %)	Pearson Chi square test
Gender	Male	138 (53.3)			
	Female	121 (46.7)			
Academic year (1 to 6th year of the medical class)	1st year	52 (20)	27 (51.9)	25 (48.1)	χ^2^(5) = 3.51, *p* = 0.622
	2nd year	75 (29)	35 (46.7)	40 (53.3)	
	3rd year	25 (9.7)	13 (52)	12 (48)	
	4th year	30 (11.6)	18 (60)	12 (40)	
	5th year	46 (17.8)	25 (54.3)	21 (45.7)	
	6th year	31 (12)	20 (64.5)	11 (35.5)	
Academic Achievement Group	Above	39 (15.1)	30 (76.9)	9 (23.1)	χ^2^(2) = 15.725, *p* = 0.00
	Average	119 (45.2)	67 (56.3)	52 (43.7)	
	Below	101 (39)	41 (40.6)	60 (59.4)	
Have you read the Student Disciplinary Regulation code for Higher Education Institutions?	Yes	28 (10.8)	16 (57.1)	12 (42.9)	χ^2^(1) = 0.188, *p* = 0.665
	No	231 (89.2)	122 (52.8)	109 (47.2)	

The scores for the whole scale and for the subscales are given in Table 2. All participants had the highest tendency for “Dishonesty in works such as assignments and projects”, with a score of 2.28, and the lowest score for “Tendency towards citation dishonesty” with a mean of 2.02 (Table 2). There was a statistically significant difference between male and female participants in the “Tendency towards academic dishonesty” values (Table 2). The highest tendency in both male and female students was observed for the factor “Dishonesty in works such as assignments and projects” (Table 2). The female participants showed the lowest tendency for the factor “Tendency towards cheating”, whereas the male participants showed the lowest tendency for the factor “Tendency towards citation dishonesty” (Table 2).

**Table 2:** The scores for the tendency towards academic dishonesty, separated by gender

Tendency Scale	Total Score (SD)	Male Score (SD)	Female Score (SD)	*P*
Tendency towards academic dishonesty				
	2.15 (0.61)	2.26 (0.65)	2.04 (0.55)	0.005*
Subscales:				
Tendency towards cheating	2.06 (0.93)	2.17 (0.97)	1.93 (0.87)	0.04*
Dishonesty in works such as assignments and projects	2.29 (0.70)	2.40 (0.75)	2.16 (0.62)	0.005*
Research and reporting process dishonesty	2.25 (0.71)	2.39 (0.74)	2.09 (0.64)	0.001*
Tendency towards citation dishonesty	2.02 (0.69)	2.07 (0.70)	1.96 (0.68)	0.213

*SD* standard deviation, * *P* < 0.05

Assessment of the tendency towards academic dishonesty with respect to academic year revealed scores varying from 2.01 to 2.42 (Table 3). Students in the first three grades displayed similar tendency scores, those in the fourth grades showed the lowest tendency, and those in the sixth grade showed the highest tendency. However, the only significant score was subscale four (Tendency towards citation dishonesty) (Table 3). In multiple comparison, the significant difference was shown between “4^th^ vs 6^th^ grade” and “5^th^ vs 6^th^ grade” (Table 3).

**Table 3:** Tendency towards academic dishonesty scores with the respect to academic year

	Class (Academic year)						
	1st	2nd	3rd	4th	5th	6th	P
Scale	Score (SD)	Score (SD)	Score (SD)	Score (SD)	Score (SD)	Score (SD)	
Tendency towards academic dishonesty	2.15 (0.60)	2.15 (0.56)	2.13 (0.44)	2.01 (0.56)	2.11 (0.78)	2.42 (0.63)	0.169
Subscales							
Tendency towards cheating	1.88 (0.94)	2.07 (0.80)	1.98 (0.82)	2.17 (0.93)	1.98 (1.01)	2.38 (1.11)	0.243
Dishonesty in works such as assignments and projects	2.37 (0.75)	2.26 (0.64)	2.26 (0.45)	2.09 (0.69)	2.24 (0.84)	2.49 (0.70)	0.319
Research and reporting process dishonesty	2.22 (0.64)	2.22 (0.68)	2.41 (0.61)	2.08 (0.71)	2.28 (0.79)	2.37 (0.84)	0.537
Tendency towards citation dishonesty	2.06 (0.71)	2.05 (0.60)	1.91 (0.59)	1.72 (0.52)	1.93 (0.84)	2.40 (0.69)	0.004*
	Multiple comparison *P*		*Mean Difference*			*95% CI* *Lower / Upper*	
Subscale 4. Tendency towards citation dishonesty	1st grade vs. 2nd grade: 1		0.015			−0.333 / 0.364	
	1st grade vs 3rd grade: 0.929		0.157			−0.313 / 0.627	
	1st grade vs 4th grade: 0.217		0.347			−0.095 / 0.790	
	1st grade vs. 5th grade: 0.933		0.129			−0.262 / 0.520	
	1st grade vs. 6th grade: 0.247		−0.334			− 0.772 / 0.104	
	2nd grade vs 3rd grade:0.942		0.142			−0.304 / 0.588	
	2nd grade vs 4th grade:0.203		0.332			−0.085 / 0.749	
	2nd grade vs 5th grade: 0.945		0.114			−0.248 / 0.476	
	2nd grade vs 6th grade: 0.150		−0.349			− 0.761 / 0.063	
	3rd grade vs 4th grade: 0.903		0.190			−0.333 / 0.713	
	3rd grade vs 5th grade: 1		−0.028			− 0.508 / 0.452	
	3rd grade vs 6th grade: 0.075		−0.491			−1.010 / 0.028	
	4th grade vs 5th grade: 0.738		−0.218			−0.671 / 0.235	
	4th grade vs 6th grade: 0.001*		−0.681			− 1.176 / -0.187	
	5th grade vs 6th grade: 0.039*		−0.463			−0.912 / -0.014	

*SD* standard deviation, * *P* < 0.05

*P < 0.05

Students who believed that they were below the class average displayed a higher tendency towards academic dishonesty than other students, for all factors and in terms of the general tendency (Table 4).

**Table 4:** Scores for the factors based on different levels of academic achievement

Academic Achievement								
	Below Score (SD)	Average Score (SD)	Above Score (SD)	*P*	Multiple comparison *P*	*Mean Difference*	*95% CI* *Lower bound / Upper bound*	
Tendency towards academic dishonesty	2.36 (0.74)	2.21 (0.60)	2.01 (0.55)	0.005*	Above vs Below: 0.007*	−0.346	−0.62	− 0.08
					Above vs Average: 0.043*	−0.198	-0.39	0.00
					Below vs Average: 0.380	0.149	−0.11	0.41
Subscales:	Below Score (SD)	Average Score (SD)	Above Score (SD)	*P*	Multiple comparison *P*			
Tendency towards cheating	2.48 (1.18)	2.10 (0.87)	1.84 (0.83)	0.001*	Above vs Below: 0.001*	−0.637	−1.04	−0.24
					Above vs Average: 0.080	−0.265	−0.55	0.02
					Below vs Average: 0.068	0.373	−0.02	0.77
Dishonesty in works such as assignments and projects	2.43 (0.80)	2.38 (0.67)	2.12 (0.67)	0.008*	Above vs Below: 0.045	−0.312	−0.62	−0.01
					Above vs Average: 0.016	−0.260	−0.48	−0.04
					Below vs Average: 0.913	0.052	−0.25	0.35
Research and reporting process dishonesty	2.50 (0.89)	2.30 (0.71)	2.10 (0.60)	0.006*	Above vs Below: 0.007*	−0.403	−0.71	−0.09
					Above vs Average: 0.081	−0.204	−0.43	0.02
					Below vs Average: 0.270	0.200	−0.10	0.50
Tendency towards citation dishonesty	2.08 (0.71)	2.04 (0.70)	1.98 (0.67)	0.659				

*SD* standard deviation, *CI* confidence interval, * *P* < 0.05

When the students were asked whether they had read the code of academic ethics published by the Higher Education Institution, the majority of them (*n* = 231, 80.2%) stated that they had no knowledge of it, and only a few of them (*n* = 28, 10.8%) stated that they had read the rules. On the other hand, whether or not the students had read the rules did not have a significant impact on their tendency towards academic dishonesty (*P* = 0.352) (Table 5).

**Table 5:** Scores for the factors on the basis of the knowledge of the code of academic ethics

	Read the code of academic ethics		
	No (*n* = 231)	Yes (*n* = 28)	*P*
Scale	Score (SD)	Score (SD)	
Tendency towards academic dishonesty	2.14 (0.62)	2.26 (0.60)	0.352
Subscales			
Tendency towards cheating	2.04 (0.90)	2.24 (1.12)	0.282
Dishonesty in works such as assignments and projects	2.29 (0.70)	2.29 (0.72)	0.993
Research and reporting process dishonesty	2.23 (0.71)	2.46 (0.66)	0.092
Tendency towards citation dishonesty	2.01 (0.68)	2.11 (0.689)	0.486

*SD* standard deviation

Despite significant results in Tables 2, 3 and 4, multivariable analysis showed no statistically significance on relationship between overall score and factors of gender, academic achievement and academic year (Table 6).

**Table 6:** Multivariable analysis for tendency towards academic dishonesty scores

	Type III Sum of Squares	df	Mean Square		F	Sig.
Corrected model	16.796*	33	0.509		1.413	0.076
Intercept	560.441	1	560.441		1556.145	0.000
Gender	0.671	1	0.671		1.862	0.174
Academic Achievement	1.878	2	0.939		2.607	0.076
Academic year	3.356	5	0.671		1.864	0.102
Gender* Academic Achievement	0.523	2	0.261		0.726	0.485
Gender* Academic year	2.302	5	0.460		1.278	0.274
Academic Achievement* Academic year	1.641	10	0.164		0.456	0.917
Gender * Academic Achievement * Academic year	2.287	8	0.286		0.794	0.609
Error	81.033	225	0.360			
Total	1301.378	259				
Corrected Total	97.829	258				
Parameter	B	*Std. Error*	*t*	*Sig.*	*95% CI*	
					*Lower bound*	*Upper bound*
Intercept	2.864	0.346	8.265	0.000	2.181	3.546
Gender						
Female	−0.103	0.797	−0.129	0.898	−1.673	1.467
Male	0					
Achievement						
Above	−0.432	0.458	− 0.942	0.347	− 1.335	0.471
Average	− 0.675	0.384	− 1.756	0.081	− 1.432	0.083
Below	0					
Academic year						
1st	−0.136	0.458	− 0.298	0.766	−1.040	0.767
2nd	−0.374	0.384	−0.973	0.331	−1.132	0.383
3rd	−0.568	0.548	−1.037	0.301	−1.648	0.511
4th	−1.082	0.438	−2.468	0.014	−1.945	−0.218
5th	0.000	0.490	0.000	1.000	−0.966	0.966
6th	0	.				

*R Squared = 0.172 (Adjusted R Squared = 0.050)

## Discussion

The results of the study show that the students had a slight tendency towards academic dishonesty. Gender, academic achievement and academic year were firstly thought to have an impact on the tendency towards academic dishonesty. However, in a multivariable analysis the statistical significance was disappeared. Another factor that whether they had read the academic ethics code had also no significant impact on the tendency scores.

Our findings show that the total TTAD scores of participating students were 2.15 ± 0.61. This score reaches up to 2.29 ± 0.70 for dishonesty in assignments and projects. According to the results of two other different studies conducted between nursing students in Turkey, academic dishonesty tendency has been found at a medium level among nursing students [17, 18].There are various studies noting the high frequency of academic dishonesty in medical faculties [7, 20–22]. For instance, in a study conducted in Canada medical students were reported to exhibit academic dishonesty at a rate of 56% [23]. According to the results of other research, more than half of 428 medical faculty students in the USA were cheating in exams; students stated that they thought that presenting an exact copy of the assignment of a successful classmate would not be wrong, and that there was no need to provide references as the basic sources of reference were open to the public [9]. These students believed that they were not doing anything wrong, as there were no clear instructions on this issue [9]. In South Korea, a study was conducted on a group of medical faculty students who prepared assignments mainly by copying information from websites [15]. Most of the participants stated that they had no knowledge of the fact that copying information from websites without providing proper references was deemed to be misconduct, or that copying the reports of classmates would pose a serious problem [15].

The results of this study show that the participants tend slightly towards academic dishonesty. When the findings of the study are compared with the findings of studies conducted in order to determine the tendency towards academic dishonesty of students studying in areas other than medicine, this is a pleasing outcome [24, 25]. On the other hand, the low level does not mean that there is no tendency towards dishonesty.

The results of this study show that the attitude towards academic dishonesty was not influenced by recorded individual factors. Also, it had no correlation with contextual factors such as awareness of the relevant ethics code. In some researches have reported that male participants display a higher level of academic dishonesty [1, 20, 26], however other research has reported no [27], or a minor, correlation between gender and academic dishonesty as our study. Peer behaviour is considered to have more influence on academic dishonesty than gender or achievement [21]. Similar to the results of our study, in other studies conducted with nursing students in Turkey, academic dishonesty tendencies have been found greatest among male students [18] and students with lower academic achievement [17]. According to our results, sixth year students had higher tendency towards academic dishonesty when compared to all other years and the multiple comparison revealed statistically significant difference between 4th and 5th years when compared to 6th year students. According to our assumption one of the reasons behind this might be that the students in their sixth year are only receiving clinical education and are not taking any formal exam. However there is not any evidence supporting this assumption.

Whatever the reason for it, it is not morally acceptable for students to resort to academic dishonesty. One should keep in mind that a medical faculty reflects society, and cannot be expected to operate independently from it. Dishonest behaviour cannot be totally eliminated without making improvements in other fields in society [7, 9–11]. Certainly, occupational skills and competencies of medical professionals, together with sound ethical practices, are among the desirable outcomes of academic education [25]. In the last decade, there have been numerous examples of the violation of academic integrity at all grades of medical faculties and at all stages of medical education, which reveals the need for efforts to prevent and solve the problem [28].

## Limitations

The first limitation was the population of the study that represent only one site in Turkey. The second limitation was the design of our study that a cross sectional study made it difficult to assess the relationship between overall scores and factors that may be related with it. The final limitation of our study was that data were collected by a self-reporting of the participants.

## Conclusion

The results of our study are indicated to students have tendency to academic dishonesty. Our findings show that the majority of the participating students had “slight tendency towards academic dishonesty”. The majority of participating students showed more tendency to dishonesty in their tasks, assignments and projects whereas the least tendency to dishonesty was towards citation. Significant differences were observed in the TTAD scores for students with gender, different academic achievements and in different academic years. However, when multivariate analysis was performed, the significance shown in the results disappeared. The findings of the study suggested that there is a need to focus on academic dishonesty in medical education.

## Data Availability

The datasets used and analyzed during the current study are available from the corresponding author on reasonable request.
